# A Peculiar Case of Idiopathic Herpes-Zoster-Like Stevens-Johnson Syndrome (SJS)

**DOI:** 10.7759/cureus.36190

**Published:** 2023-03-15

**Authors:** Desiree E Ojo, Farah Tiab, David Walden, Seth Bernacki, Mariel Bagley

**Affiliations:** 1 Osteopathic Medicine, University of Incarnate Word School of Osteopathic Medicine, San Antonio, USA; 2 Family Medicine, Christus Santa Rosa Health System, San Antonio, USA; 3 Dermatology, Philadelphia College of Osteopathic Medicine, San Antonio, USA

**Keywords:** acyclovir therapy, kidney transplant recipients, immuno suppresion, herpes zoster virus, stevens-johnson syndrome (sjs), pathology derm

## Abstract

Epidermal necrolysis is a severe dermatological condition usually associated with adverse drug reactions involving the mucosa. Stevens-Johnson syndrome (SJS) is clinically diagnosed when an epidermal detachment of less than 10% of body surface area (BSA) is involved. In contrast, toxic epidermal necrolysis (TEN) is characterized when there is an epidermal detachment of more than 30% BSA. Epidermal necrolysis can be described as ulcerated, painful, and erythematous lesions typically appearing on the skin. Typical clinical presentations of SJS include epidermal detachment of less than 10% of BSA and mucosal involvement with prodromal flu-like symptoms. Atypical presentations of focal epidermal necrolysis include the presence of lesions in a dermatomal pattern, associated itching, and idiopathic cause. We report a rare case of suspected herpes-zoster virus (HZV)-like SJS with negative HZV serum PCR and negative varicella-zoster virus (VZV) biopsy immunostaining. This rare case of SJS was resolved with the administration of IV acyclovir and Benadryl.

## Introduction

Stevens-Johnson syndrome (SJS) is a severe and life-threatening mucocutaneous illness that is typically triggered by a drug, infection, or an idiopathic cause [1]. Clinical presentation includes a prodrome of a loss of appetite, fever, and pharyngitis. SJS is known for its characteristic painful, erythematous rash that starts off as large bullous lesions followed by dermo-epidermal detachment affecting skin, eyes, genitalia, and mouth [2]. SJS represents the less severe version of epidermal necrolysis, with toxic epidermal necrolysis (TEN) representing a more severe version of the illness [3]. TEN and SJS were first coined by Alan Lyell in 1956 [4]. Lyell characterized TEN and SJS based on the percentage of body surface area (BSA) affected. SJS involves less than 10% of BSA and TEN involves more than 30% of BSA [4]. In unique cases, SJS/TEN can exist when 10%-30% of BSA is affected [4]. SJS has an estimated incidence rate of 1.2 to 6 per million people worldwide annually, in which 10%-20% of cases occur in ages one to three years old [2,3] and is most commonly found in women and the elderly [3]. The mechanism of SJS is not fully understood; but it is postulated that it is caused by CD8^+^ cell exocytosis of apoptotic enzymes, activation of the Fas ligand pathway, and tumor necrosis factor-alpha death receptor pathway that leads to apoptosis of keratinocytes [3].

## Case presentation

A 42-year-old female with a medical history of immunodeficiency, nonspecific autoimmune glomerulonephritis, bilateral kidney transplant, antiphospholipid syndrome, anxiety, depression, and hyperlipidemia presented to the emergency department (ED) with the chief concern of a diffuse painful rash that the patient stated felt “like chicken pox” with accompanied flu-like symptoms. She takes immunosuppressive medications due to her history of a kidney transplant, including tacrolimus 2mg oral once in the morning, 1.5 mg oral nightly, and mycophenolate mofetil 500mg oral twice daily. Additional medications include apixaban 5mg oral twice daily, atorvastatin 40 mg oral once daily, hydrocortisone 10 mg oral once daily, pantoprazole 40 mg oral twice daily, alprazolam 1 mg oral PRN and venlafaxine XL 150 mg oral once daily. She reported 10 days prior, that she began to notice itchy papules on her face which subsequently spread to her trunk and extremities. As the lesions spread, they began to form painful pustules that progressed to shallow, crusted ulcerations in a dermatomal pattern. She described a painful pressure-like sensation on her backside and “shooting” sensations felt down her legs. She also endorsed fevers with a maximum temperature of 101 degrees Fahrenheit, intermittent chills, body aches, and extreme muscle weakness. She experienced intermittent nausea and numerous bouts of vomiting with associated diarrhea. She denied recent travel or exposure to other people with rashes or flu-like symptoms. She reported that she has been with the same sexual partner for the past 10 years and does not have any concerns about sexually transmitted infections (STIs). She denied any visual changes, earaches, mouth blisters, chest pain, cough, shortness of breath, leg pain, peripheral edema, or arthralgias.

**Figure 1:**
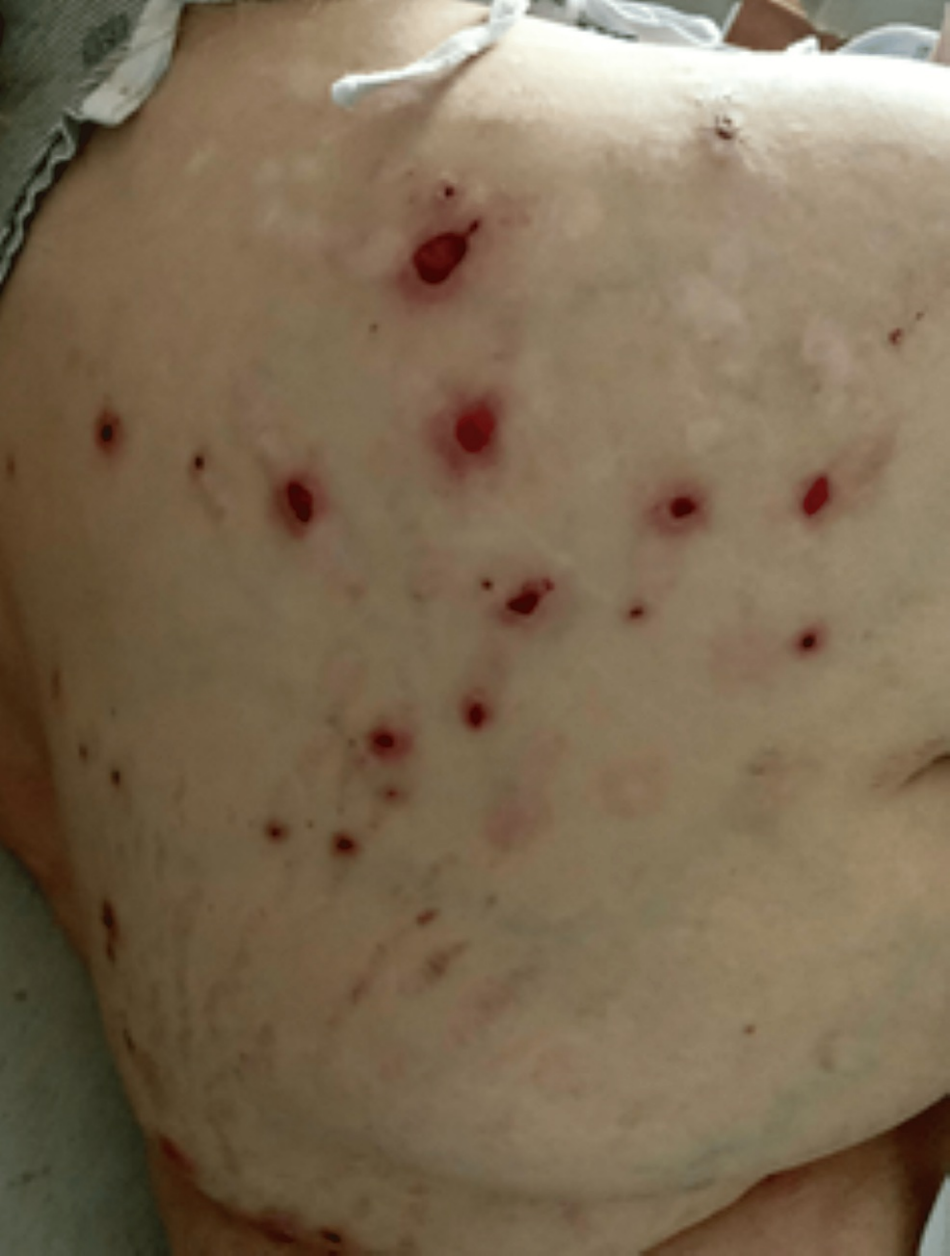
Right upper back displaying erythematous and ulcerated rash on Day 16

**Figure 2:**
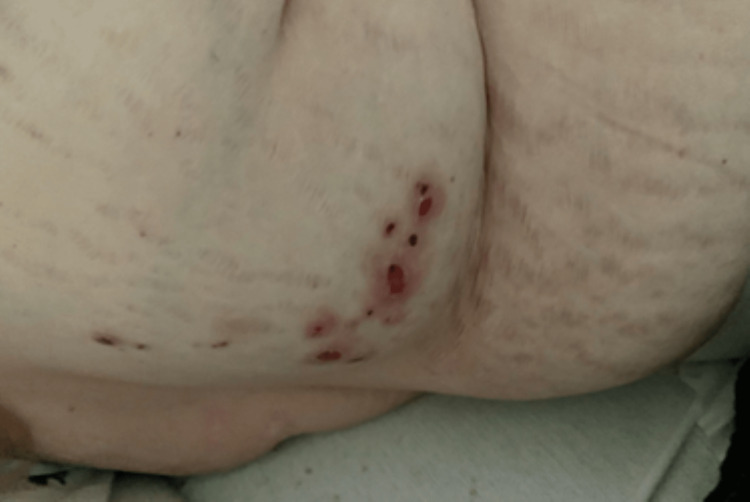
Right lower back displaying rash in coalesced and dermatomal pattern on Day 16

Based on the patient’s history, clinical picture, and physical examination, a working diagnosis of herpes-zoster virus (HZV) was made by the chief family medicine resident on-call and the infectious disease consultation team at the time of admission. The patient was given Benadryl 50 mg IV once and acyclovir 900 mg IV once in the emergency department and was admitted to the hospital for possible monkeypox vs. disseminated herpes simplex virus vs. HZV. She was treated with Benadryl 50 mg IV, gabapentin 400mg oral and acyclovir 900 mg IV with clinical improvement of her symptoms. Serological test results for *Treponema pallidum* serum antibodies, SARS-CoV-2 PCR swab, cytomegalovirus (CMV) DNA serum PCR, herpes simplex virus (HSV) I/II DNA serum PCR, Influenza Type A/B PCR swab, orthomyxovirus serum PCR and varicella-zoster virus (VZV) serum PCR, were collected in the ED prior to treatment. All tests came back negative. A 3-mm punch biopsy was taken from the right lateral thoracolumbar region one week before discharge, which revealed ulcers with associated resolving folliculitis and focal epidermal necrosis. Immunostaining for HSV-1, HSV-2, VZV, and fungal PAS stain were negative. Neither diagnostic inclusions of monkeypox (mpox) virus nor malignancy were identified (Figures 1, 2).

After more than one month in the hospital, the patient was discharged. Two weeks post discharge, the patient was seen once in the clinic for a follow-up appointment. Her condition completely resolved with no reoccurrence.

## Discussion

SJS is a life-threatening hypersensitivity reaction usually caused by medications, but can occasionally occur due to infectious agents, vaccines, and even certain foods [5-7]. A study by Roujeau et al. in 1995 found an association between corticosteroid use and increased risk of SJS, which is counterintuitive given that SJS is likely immune-mediated, and corticosteroids are known to facilitate immunosuppression [7]. This could indicate an association between drug-induced immunosuppression as seen in our patient and SJS. However, the onset of SJS is between 7 and 47 days after the initiation of the offending medication, and our patient had been taking these medications for months to years. In addition, SJS can occur at any level of immunosuppression and from over a hundred different medications [6]. The most common medications that cause SJS include NSAIDS, phenobarbital, carbamazepine, phenytoin, erythromycin, cefotaxime, TMP-SMX, allopurinol, amoxicillin, and cloxacillin [2]. The most common infections that cause SJS include *M. pneumoniae, Rickettsia, Mycobacterium species,* cytomegalovirus, herpesvirus, coxsackievirus, parvovirus, and influenza virus [2]. Family history and certain HLA allotypes can predispose certain individuals to develop SJS as well [8]. This case report illustrates the need for reliable in-office tests, such as breath biomarkers, to more easily diagnose SJS due to its morbidity and ability to mimic other conditions [2]. The list of differential diagnoses is extensive and includes other severe cutaneous adverse reactions (SCAR): erythema multiforme major, staphylococcal scalded skin syndrome, pemphigus vulgaris, and pemphigus foliaceus among others [8]. The use of exhaled breath biomarkers has been postulated as a potential solution for early detection [1].

By diagnosis of exclusion and the result of the pathologist report, we conclude that idiopathic SJS is the most probable diagnosis for our patient. Our team acknowledges that the clinical presentation of this patient suggests HZV infection due to clinical improvement with acyclovir and Benadryl. It is important to consider possible false negative results from the PCR analysis. Studies suggest that in order to achieve optimal sensitivity in the diagnosis of HZV the combination of PCR and serology of serum samples must be completed, as PCR is most sensitive in the first days of the illness [9]. Our case also highlights the need for increased sensitivity of PCR and serology tests, especially in clinical presentations suggestive of HZV infection with previously negative serum PCR results.

## Conclusions

Future studies should focus on identifying exhaled breath biomarkers unique to SJS and genetic testing to offer insight into individuals more prone to SJS. Atypical presentations of SJS can result in a delay in diagnosis and can contribute to poor outcomes for patients. SJS has a disproportionately high mortality rate with most deaths occurring due to sepsis and multiorgan failure. With an estimated incidence rate of 1.2 to 6 per million people annually worldwide, further studies should be conducted to evaluate cost-effective methods to properly identify and diagnose unique presentations of SJS in the general population.

## References

[1] Corradi M, Zinelli C, Caffarelli C (2007). Exhaled breath biomarkers in asthmatic children. Inflamm Allergy Drug Targets.

[2] De Guido C, Calderaro A, Ruozi MB (2020). An unusual cause of Steven-Johnson syndrome. Acta Biomed.

[3] Nunes AL, Simoes L, Figueiredo C, Carvalho R, Lima J, Santos RM (2022). Toxic epidermal necrolysis-like lupus erythematous presentation following SARS-CoV-2 infection. J Med Cases.

[4] Pathania V, Baveja S, Sinha A, Bhatia JK (2022). An atypical presentation of toxic epidermal necrolysis without mucosal involvement. Med J Armed Forces India.

[5] Schwartz RA, McDonough PH, Lee BW (2013). Toxic epidermal necrolysis: Part I. Introduction, history, classification, clinical features, systemic manifestations, etiology, and immunopathogenesis. J Am Acad Dermatol.

[6] Dziuban EJ, Hughey AB, Stewart DA, Blank DA, Kochelani D, Draper HR, Schutze GE (2013). Stevens-Johnson syndrome and HIV in children in Swaziland. Pediatr Infect Dis J.

[7] Roujeau JC, Kelly JP, Naldi L (1995). Medication use and the risk of Stevens-Johnson syndrome or toxic epidermal necrolysis. N Engl J Med.

[8] Li Y, Chu Y, Li Q, Li L, Tuo Y, Wang H, Zhang L (2023). Clinical features and management of stevens-johnson syndrome and toxic epidermal necrolysis: a retrospective single-center study [PREPRINT]. Dermatitis.

[9] Dobec M, Bossart W, Kaeppeli F, Mueller-Schoop J (2008). Serology and serum DNA detection in shingles. Swiss Med Wkly.

